# A practical nomogram and risk stratification system for predicting survival outcomes in neuroblastoma patients: a SEER population-based study

**DOI:** 10.1007/s00432-023-05110-5

**Published:** 2023-07-11

**Authors:** Xiaoyu Zhuo, Liangfeng Xia, Wenjing Tang, Wenqi He

**Affiliations:** 1 Department of Pediatric Hematology and Oncology, Chengdu Women’s and Children’s Central Hospital, School of Medicine, University of Electronic Science and Technology of China, Chengdu, 611731 China; 2 Department of Pediatric Surgery, Chengdu Women’s and Children’s Central Hospital, School of Medicine, University of Electronic Science and Technology of China, Chengdu, 611731 China

**Keywords:** Neuroblastoma, Nomogram, Prognosis, Risk stratification system

## Abstract

**Background:**

Neuroblastoma (NB) is a childhood malignancy with marked heterogeneity, resulting in highly variable outcomes among patients. This study aims to establish a novel nomogram and risk stratification system to predict the overall survival (OS) for patients with NB.

**Methods:**

We analyzed neuroblastoma patients from the Surveillance, Epidemiology, and End Results (SEER) database between 2004 and 2015. The nomogram was constructed using independent risk factors for OS, identified through univariate and multivariate Cox regression analyses. The accuracy of this nomogram was evaluated with the concordance index, receiver operating characteristic curve, calibration curve, and decision curve analysis. In addition, we developed a risk stratification system based on the total score of each patient in the nomogram.

**Results:**

A total of 2185 patients were randomly assigned to the training group and the testing group. Six risk factors, including age, chemotherapy, brain metastases, primary site, tumor stage, and tumor size, were identified in the training group. Using these factors, a nomogram was constructed to predict 1-, 3-, and 5-year OS of NB patients. This model exhibited superior accuracy in the training and testing groups, exceeding traditional tumor stage prediction. Subgroup analysis suggested worse prognosis for retroperitoneal origin in the intermediate-risk group and adrenal gland origin in the high-risk group compared to other sites. Additionally, the prognosis for high-risk patients significantly improved after surgery. We also developed a web application to make the nomogram more user-friendly in clinical practices.

**Conclusion:**

This nomogram demonstrates excellent accuracy and reliability, offering more precise personalized prognostic predictions to clinical patients.

## Introduction

Neuroblastoma (NB) is a childhood malignancy that originates from the developing sympathetic nervous system (Maris 2010; Tolbert and Matthay 2018; Vo et al. 2014; Irwin and Park 2015). The most common primary sites of NB are the adrenal medulla and the sympathetic ganglia (Maris 2010; Tolbert and Matthay 2018; Vo et al. 2014; Irwin and Park 2015). As the most common extracranial solid tumor in childhood, NB accounts for approximately 6–10% of all pediatric malignancies, with an annual incidence of around 1 case per 10,000 children under the age of 15 in the United States (Lu et al. 2021; Yao et al. 2017). The treatment of neuroblastoma follows a multidisciplinary model, determined by a multitude of factors including age at diagnosis, tumor stage, and tumor biology. In recent years, the ongoing enhancement of conventional treatment techniques like surgery, chemotherapy, and radiotherapy, combined with the advancement of emerging therapies such as immunotherapy, has substantially improved the prognosis for children with neuroblastoma.

Despite these advancements, the prognosis for patients with neuroblastoma still demonstrates substantial variability due to the considerable tumor heterogeneity. Patients with low- and intermediate-risk neuroblastoma exhibit an overall survival (OS) rate exceeding 90%, while those with high-risk neuroblastoma face a dismal prognosis, with survival rates as low as 50% (Baker et al. 2010; Rubie et al. 2011; Strother et al. 2012; Pinto et al. 2015; Morgenstern et al. 2018). The prognosis of neuroblastoma is widely recognized as highly reliant on the tumor stage, with two primary staging systems in use: one centered on post-surgical staging (the International Neuroblastoma Staging System, INSS), and the other emphasizing risk classification prior to treatment (the International Neuroblastoma Risk Group Staging System, INRGSS) (Brodeur et al. 1993; McCarville 2011; Monclair et al. 2009). However, current tumor stage approaches fail to provide precise personalized prognostic models for predicting OS in patients with neuroblastoma. Consequently, there is an urgent need to assess the prognosis and risk stratification of patients with neuroblastoma early on.

In recent years, nomograms have demonstrated superiority over traditional TNM staging system and have been extensively used for individualized estimation of prognosis in various malignancies (Liang et al. 2015; Zhou et al. 2018; Sharouni et al. 2021). Within the realm of neuroblastoma prognosis, nomograms exhibit immense potential in offering a personalized approach to OS and risk stratification, generating a visually interpretable probability of a specific outcome. The objective of this study was to develop an accurate and reliable predictive nomogram for estimating OS and providing individualized risk assessment for neuroblastoma patients using the Surveillance, Epidemiology, and End Results (SEER) database.

## Methods

### Patients and methods

#### Data source

The patient data were extracted from the SEER database of the National Cancer Institute (NCI). The SEER database, administered by the NCI, serves as the authoritative source of information that provides updated data on cancer incidence and patient survival rates from population-based cancer registries, covering approximately 48.0% of the U.S. population.

#### Patients selection

**Fig. 1:**
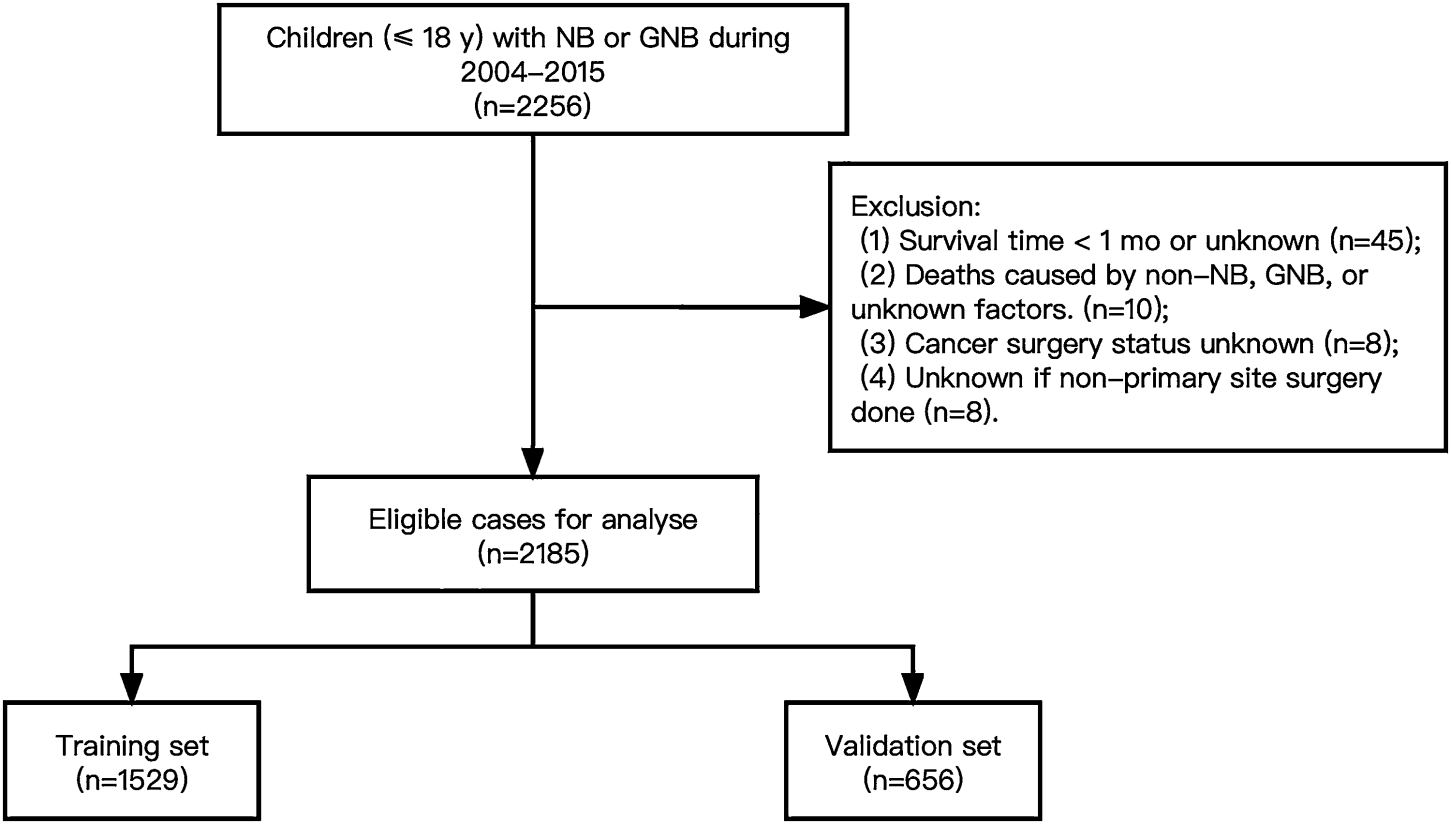
Flowchart illustrating the inclusion and exclusion of patients

Patients with neuroblastoma between 2004 and 2015 were selected from the SEER database according to the International Classification of Diseases for Oncology 3rd Edition (ICD-O-3). Inclusion criteria were: (1) age equal to or below 18 years, and (2) diagnosed with neuroblastoma or ganglioneuroblastoma (GNB). Exclusion criteria included: (1) survival time less than 1 month or unknown, (2) deaths attributed to causes other than neuroblastoma or ganglioneuroblastoma or unknown cause of death, (3) uncertainty surrounding whether cancer-directed surgery was performed, and (4) uncertainty regarding whether a surgical procedure other than at the primary site was performed. The selection criteria and screening process are depicted in Fig. 1.

#### Clinical variables and outcomes

The collected patient information included age, race, sex, histology, primary site, tumor number, tumor size, first malignant primary indicator, tumor grade, distant metastases, tumor stage, surgery, scope of regional lymph node surgery, regional nodes, surgical procedure of other sites, chemotherapy, and radiotherapy. Racial categories were classified as White and others (including Asian or Pacific Islander, American Indian/Alaska Native, Black, or Unknown). The primary site was determined in several locations including the adrenal gland, retroperitoneum, and others. The tumor stage was classified into four types based on SEER Combined Summary Stage: localized, regional, distant, and unknown/unstaged. Distant metastases occurred in organs such as the bone, brain, liver, and lung. The optimal cutoff value for tumor size was determined by the X-Tile software, and then categories as 0–62 mm, 63–87 mm, 88–989 mm, and unknown. Tumor grade was stratified as Grade I (well-differentiated), Grade II (moderately differentiated), Grade III (poorly differentiated), Grade IV (undifferentiated), or unknown. OS served as the primary endpoint, defined as the duration (in months) from the date of diagnosis to death or the last follow-up.

### Statistical analysis

Analyses were performed using SPSS 26.0 (IBM, Chicago, IL, USA) and R software (version 4.2.2). Statistical significance was defined as *P* < 0.05 using two-sided tests. The measurement data was described using the median and interquartile range (IQR). The enumeration data, described as the number of cases or percentage, was analyzed using the chi-square test. A total of 2185 patients were assigned to training and testing groups in a 7:3 ratio. The training group was designated for constructing the nomogram and internal validation, while the testing group was used for model validation.

#### Prognostic nomogram construction

Univariate and multivariate Cox regression analyses were carried out to identify independent prognostic factors. Significant factors (*P* < 0.05) were selected for nomogram construction. Variables were represented as line segments with varying lengths according to weight, with scores ranging from 0 to 100. Total scores predicted 1, 3, and 5-year OS.

#### Prognostic nomogram validation

The concordance index (C-index) was used to measure the accuracy of model predictions. A value above 0.7 indicates that the predictive model has excellent discriminative ability. The receiver operating characteristic (ROC) curve was employed to evaluate the performance of classification models. An area under the ROC curve (AUC) above 0.7 signifies that the model possesses excellent discriminative ability. The calibration curve was used to verify the accuracy of probability predictions. A curve close to the 45-degree diagonal line indicates that the predicted probabilities are consistent with the actual observed probabilities. Finally, decision curve analysis (DCA) was utilized to appraise the clinical utility of a predictive model at different thresholds.

#### Risk stratification based on nomogram

Patients were categorized into three groups according to their total scores on the nomogram using X-tile software: the low-risk group (total score ≤ 140), the intermediate-risk group (140 < total score < 223), and the high-risk group (total score ≥ 223). The differences in survival among these risk categories were compared using Kaplan–Meier curves and log-rank tests.

#### Dynamic nomogram construction

A web-based dynamic nomogram was constructed using the open source R Shiny Server, which allows clinicians to conveniently assess patient prognosis using the nomogram.

## Results

### Patient characteristics

A total of 2,185 patients were diagnosed with neuroblastoma or ganglioneuroblastoma between 2004 and 2015. Of these, 1529 were assigned to the training group and 656 to the testing group. The demographic and clinical features of the patients are outlined in Table 1. No significant differences were observed between the training and testing groups (*P* > 0.05). Generally, the median age of children was 1 year (IQR: 0–3), comprising 1146 males and 1039 females. The primary tumor site was primarily the adrenal gland (45.9%), with 10.3% in the retroperitoneum. Bone, liver, lung, and brain metastases were present in 16.3%, 6.0%, 2.4%, and 1.8% of the patients, respectively. Moreover, 78.8% of patients underwent surgery, 66.7% received chemotherapy, and 24.4% underwent radiotherapy.

**Table 1:** Demographics and clinical characteristics of patients with neuroblastoma in the training and testing groups

	Train, N = 1529 (%)	Test, N = 656 (%)	All, N = 2185 (%)	t/χ^2^ (*P*)
Age (IQR)	1 (0–3)	1 (0–3)	1 (0–3)	0.333 (0.739)
Race				0.117 (0.732)
Other	367 (24.0)	153 (23.3)	520 (23.8)	
White	1162 (76.0)	503 (76.7)	1665 (76.2)	
Sex				3.174 (0.075)
Male	821 (53.7)	325 (49.5)	1146 (52.4)	
Female	708 (46.3)	331 (50.5)	1039 (47.6)	
Histology				0.249 (0.617)
Neuroblastoma	1271 (83.1)	551 (84.0)	1822 (83.4)	
Ganglioneuroblastoma	258 (16.9)	105 (16.0)	363 (16.6)	
Primary site				0.814 (0.666)
Other	661 (43.2)	297 (45.3)	958 (43.8)	
AdrenalGland	710 (46.4)	292 (44.5)	1002 (45.9)	
Retroperitoneum	158 (10.3)	67 (10.2)	225 (10.3)	
Tumor number				0 (0.997)
> 1	35 (2.3)	15 (2.3)	50 (2.3)	
1	1494 (97.7)	641 (97.7)	2135 (97.7)	
Tumor size (mm)				3.899 (0.273)
0–62	567 (37.1)	265 (40.4)	832 (38.1)	
63–87	259 (16.9)	119 (18.1)	378 (17.3)	
88–989	372 (24.3)	147 (22.4)	519 (23.8)	
Unknown	331 (21.6)	125 (19.1)	456 (20.9)	
First malignant primary indicator				0.965 (0.326)
No	16 (1.0)	4 (0.6)	20 (0.9)	
Yes	1513 (99.0)	652 (99.4)	2165 (99.1)	
Tumor grade				0.444 (0.931)
I/II	32 (2.1)	13 (2.0)	45 (2.1)	
III	683 (44.7)	285 (43.4)	968 (44.3)	
IV	115 (7.5)	48 (7.3)	163 (7.5)	
Unknown	699 (45.7)	310 (47.3)	1009 (46.2)	
Bone metastases				0.293 (0.864)
No	476 (31.1)	201 (30.6)	677 (31.0)	
Unknown	800 (52.3)	351 (53.5)	1151 (52.7)	
Yes	253 (16.5)	104 (15.9)	357 (16.3)	
Brain metastases				1.909 (0.385)
No	693 (45.3)	293 (44.7)	986 (45.1)	
Unknown	805 (52.6)	355 (54.1)	1160 (53.1)	
Yes	31 (2.0)	8 (1.2)	39 (1.8)	
Liver metastases				0.515 (0.773)
No	634 (41.5)	262 (39.9)	896 (41.0)	
Unknown	802 (52.5)	355 (54.1)	1157 (53.0)	
Yes	93 (6.1)	39 (5.9)	132 (6.0)	
Lung metastases				0.486 (0.784)
No	689 (45.1)	285 (43.4)	974 (44.6)	
Unknown	804 (52.6)	355 (54.1)	1159 (53.0)	
Yes	36 (2.4)	16 (2.4)	52 (2.4)	
Tumor stage (Combined Summary Stage)				4.366 (0.225)
Distant	742 (48.5)	319 (48.6)	1061 (48.6)	
Localized	385 (25.2)	142 (21.6)	527 (24.1)	
Regional	333 (21.8)	161 (24.5)	494 (22.6)	
Unknown/Unstaged	69 (4.5)	34 (5.2)	103 (4.7)	
Surgery				1.306 (0.253)
Yes	1195 (78.2)	527 (80.3)	1722 (78.8)	
No	334 (21.8)	129 (19.7)	463 (21.2)	
Scope of regional lymph node surgery				4.431 (0.218)
> 4	168 (11.0)	83 (12.7)	251 (11.5)	
1–3	284 (18.6)	125 (19.1)	409 (18.7)	
None	876 (57.3)	347 (52.9)	1223 (56.0)	
Other	201 (13.1)	101 (15.4)	302 (13.8)	
Regional nodes				4.677 (0.197)
Negative	147 (9.6)	80 (12.2)	227 (10.4)	
None	822 (53.8)	328 (50.0)	1150 (52.6)	
Positive	407 (26.6)	185 (28.2)	592 (27.1)	
Unknown	153 (10.0)	63 (9.6)	216 (9.9)	
Surgical procedure of other site				0.108 (0.742)
None	1373 (89.8)	586 (89.3)	1959 (89.7)	
Other	156 (10.2)	70 (10.7)	226 (10.3)	
Chemotherapy				0.220 (0.639)
No/Unknown	504 (33.0)	223 (34.0)	727 (33.3)	
Yes	1025 (67.0)	433 (66.0)	1458 (66.7)	
Radiotherapy				1.105 (0.293)
Yes	364 (23.8)	170 (25.9)	534 (24.4)	
None/Unknown	1165 (76.2)	486 (74.1)	1651 (75.6)	

### Nomogram construction

**Fig. 2:**
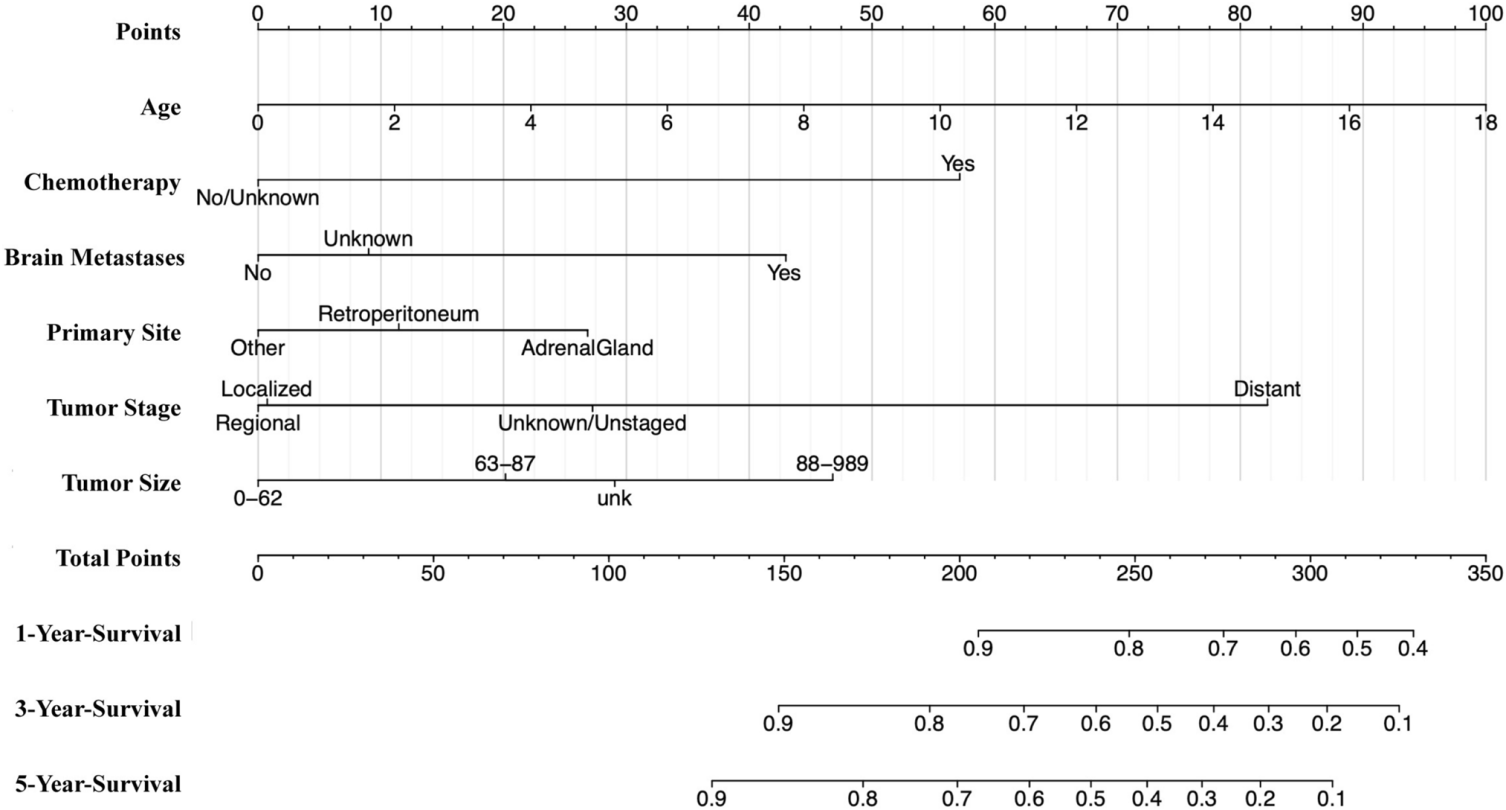
Nomogram predicting 1-, 3-, and 5-year overall survival (OS) rates for patients with neuroblastoma

We initially identified variables strongly associated with outcomes (*P* < 0.05) through univariate analysis in the training group. These variables comprised 14 factors, including age, histology, metastases (bone, brain, liver, lung), regional nodes, primary tumor site, surgical procedure at other sites, tumor stage, tumor size, chemotherapy, radiation, and regional lymph node surgery, which significantly impacted OS (Table 2). These factors were incorporated into a multivariate Cox analysis for OS. Age, chemotherapy, brain metastases, primary site, tumor stage, and tumor size were identified as independent risk factors (Table 2). These factors were then used to construct a nomogram predicting 1-, 3-, and 5-year OS of NB patients (Fig. 2). The sum of scores for individual factors in the nomogram provides an estimate of patient prognosis.

**Table 2:** Univariate and multivariate Cox regression analyses of overall survival in patients with neuroblastoma in the training group

	Univariate			Multivariate		
	HR	95% CI	Z (*P*)	HR	95% CI	Z (*P*)
Age	1.09	1.07–1.12	6.8343 (< 0.001)	1.11	1.07–1.14	6.2529 (< 0.001)
Race						
Others	1.24	0.97–1.59	1.7309 (0.0835)	NA	NA	NA
White	Ref	NA	NA	Ref	NA	NA
Sex						
Male	1.21	0.96–1.51	1.6224 (0.1047)	NA	NA	NA
Female	Ref	NA	NA	Ref	NA	NA
Histology						
Neuroblastoma	Ref	NA	NA	Ref	NA	NA
Ganglioneuroblastoma	0.47	0.32–0.7	– 3.7610 (< 0.001)	0.89	0.58–1.35	– 0.5583 (0.5766)
Primary site						
Other	0.36	0.28–0.47	– 7.5319 (< 0.001)	0.61	0.46–0.82	– 3.3684 (< 0.001)
AdrenalGland	Ref	NA	NA	Ref	NA	NA
Retroperitoneum	0.72	0.5–1.04	– 1.7606 (0.0783)	0.74	0.51–1.08	– 1.5491 (0.1213)
Tumor number						
> 1	1.32	0.7–2.48	0.8611 (0.3892)	NA	NA	NA
1	Ref	NA	NA	Ref	NA	NA
Tumor size (mm)						
0–62	Ref	NA	NA	Ref	NA	NA
63–87	1.83	1.25–2.69	3.0793 (< 0.05)	1.41	0.96–2.09	1.739 (0.082)
88–989	4.05	2.97–5.52	8.8612 (< 0.001)	2.29	1.65–3.18	4.9668 (< 0.001)
Unknown	2.27	1.61–3.21	4.6807 (< 0.001)	1.57	1.1–2.23	2.481 (< 0.05)
First malignant primary indicator						
No	1.19	0.44–3.19	0.3458 (0.7295)	NA	NA	NA
Yes	Ref	NA	NA	Ref	NA	NA
Tumor grade						
I	Ref	NA	NA	Ref	NA	NA
II	1	0–Inf	0 (1)	NA	NA	NA
III	10,579,728.65	0–Inf	0.0121 (0.9903)	NA	NA	NA
IV	30,420,541.64	0–Inf	0.0129 (0.9897)	NA	NA	NA
Unknown	9,559,315.31	0–Inf	0.012 (0.9904)	NA	NA	NA
Bone metastases						
No	Ref	NA	NA	Ref	NA	NA
Unknown	1.86	1.36–2.55	3.8471 (< 0.001)	NA	NA	NA
Yes	3.33	2.33–4.74	6.6483 (< 0.001)	0.78	0.53–1.17	– 1.1881 (0.2348)
Brain metastases						
No	Ref	NA	NA	Ref	NA	NA
Unknown	1.16	0.91–1.47	1.2119 (0.2256)	0.52	0.07–3.78	– 0.6426 (0.5205)
Yes	3.61	2.11–6.19	4.6674 (< 0.001)	2.24	1.27–3.96	2.7747 (< 0.05)
Liver metastases						
No	Ref	NA	NA	Ref	NA	NA
Unknown	1.16	0.9–1.48	1.1602 (0.246)	0	0– Inf	– 0.01 (0.992)
Yes	1.78	1.15–2.75	2.5835 (< 0.05)	1.08	0.67–1.73	0.3202 (0.7488)
Lung metastases						
No	Ref	NA	NA	Ref	NA	NA
Unknown	1.16	0.92–1.47	1.2339 (0.2172)	0.81	0.11–5.94	– 0.2034 (0.8388)
Yes	3.1	1.81–5.31	4.1124 (< 0.001)	1.23	0.69–2.18	0.7024 (0.4824)
Tumor stage (Combined Summary Stage)						
Distant	Ref	NA	NA	Ref	NA	NA
Localized	0.09	0.06–0.16	– 8.9020 (< 0.001)	0.26	0.14–0.48	– 4.3176 (< 0.001)
Regional	0.15	0.1–0.23	– 8.5080 (< 0.001)	0.22	0.14–0.35	– 6.2696 (< 0.001)
Unknown/Unstaged	0.22	0.1–0.49	– 3.6779 (< 0.001)	0.37	0.15–0.93	– 2.1162 (< 0.05)
Surgery						
Yes	0.96	0.74–1.26	– 0.2664 (0.7899)	NA	NA	NA
No	Ref	NA	NA	Ref	NA	NA
Scope of regional lymph node surgery						
> 4	1.42	1.01–2	2.0423 (< 0.05)	0.74	0.44–1.26	– 1.1098 (0.2671)
1–3	1.02	0.75–1.39	0.1480 (0.8823)	0.63	0.39–1.02	– 1.8729 (0.0611)
None	Ref	NA	NA	Ref	NA	NA
Other	1.31	0.95–1.81	1.6690 (0.0951)	1.19	0.78–1.81	0.7939 (0.4272)
Regional Nodes						
Negative	Ref	NA	NA	Ref	NA	NA
None	1.35	0.86–2.1	1.3063 (0.1915)	0.85	0.49–1.49	– 0.5641 (0.5727)
Positive	1.88	1.19–2.98	2.6920 (< 0.05)	1.27	0.78–2.05	0.9701 (0.332)
Unknown	1.13	0.64–2.01	0.4305 (0.6668)	0.85	0.44–1.64	– 0.4717 (0.6371)
Surgical procedure of other site						
None	Ref	NA	NA	Ref	NA	NA
Other	1.96	1.45–2.63	4.4437 (< 0.001)	1.27	0.94–1.71	1.5311 (0.1257)
Chemotherapy						
No/Unknown	0.13	0.08–0.2	– 8.9880 (< 0.001)	0.36	0.21–0.62	– 3.6544 (< 0.001)
Yes	Ref	NA	NA	Ref	NA	NA
Radiation						
Yes	2.55	2.04–3.2	8.1488 (< 0.001)	0.95	0.74–1.22	– 0.4071 (0.684)
None/Unknown	Ref	NA	NA	Ref	NA	NA

*NA* not applicable, *Ref* reference, *Inf* infinite

### Nomograms validation

**Fig. 3:**
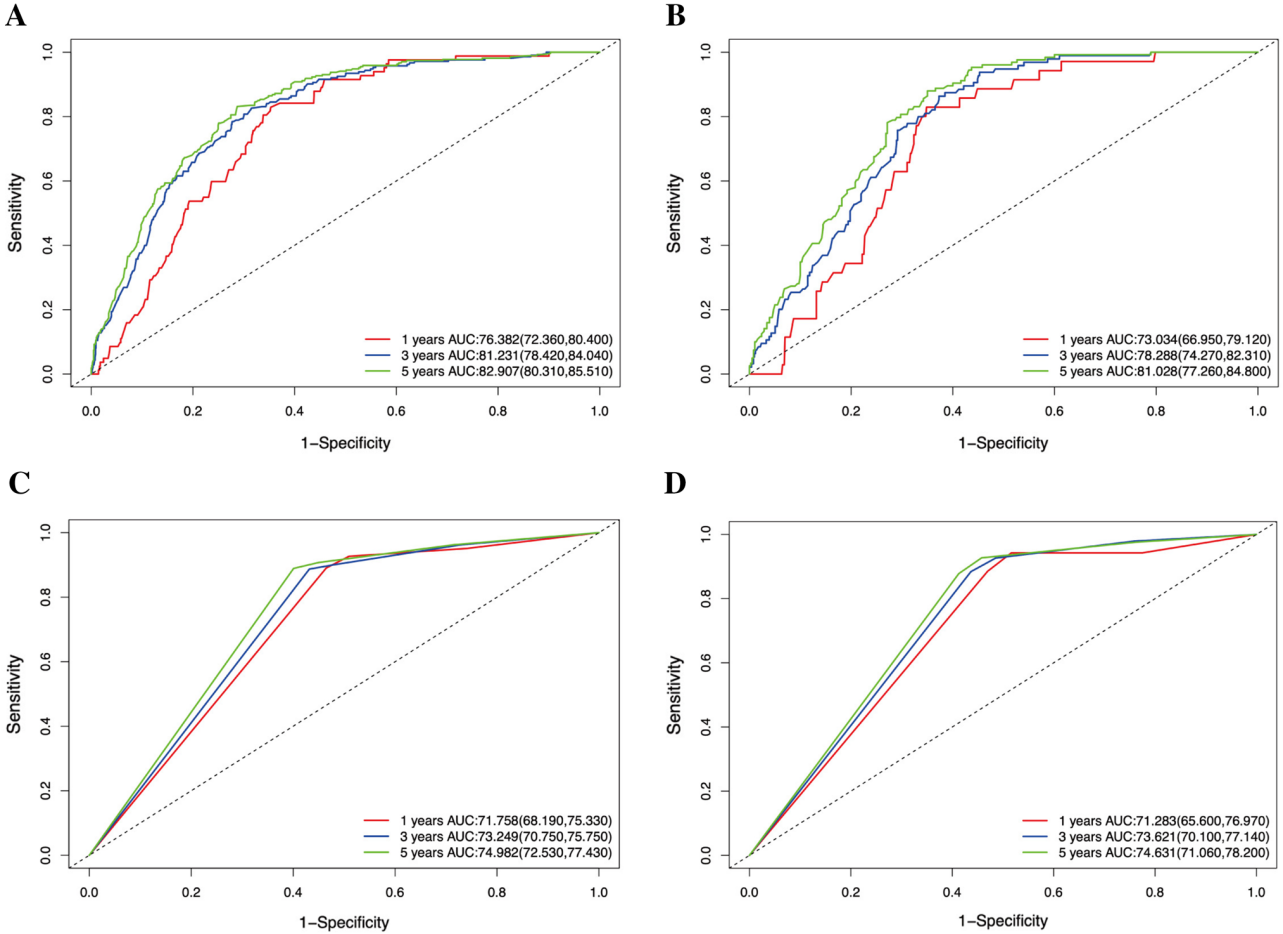
Receiver operating characteristic (ROC) curves of the nomogram and combined summary stage. **A** ROC curves for 1-, 3-, and 5-year OS using the nomogram model in the training group. **B** ROC curves for 1-, 3-, and 5-year OS using the nomogram model in the testing group. **C** ROC curves for 1-, 3-, and 5-year OS using the combined summary stage in the training group. **D** ROC curves for 1-, 3-, and 5-year OS using the combined summary stage in the testing group.

**Fig. 4:**
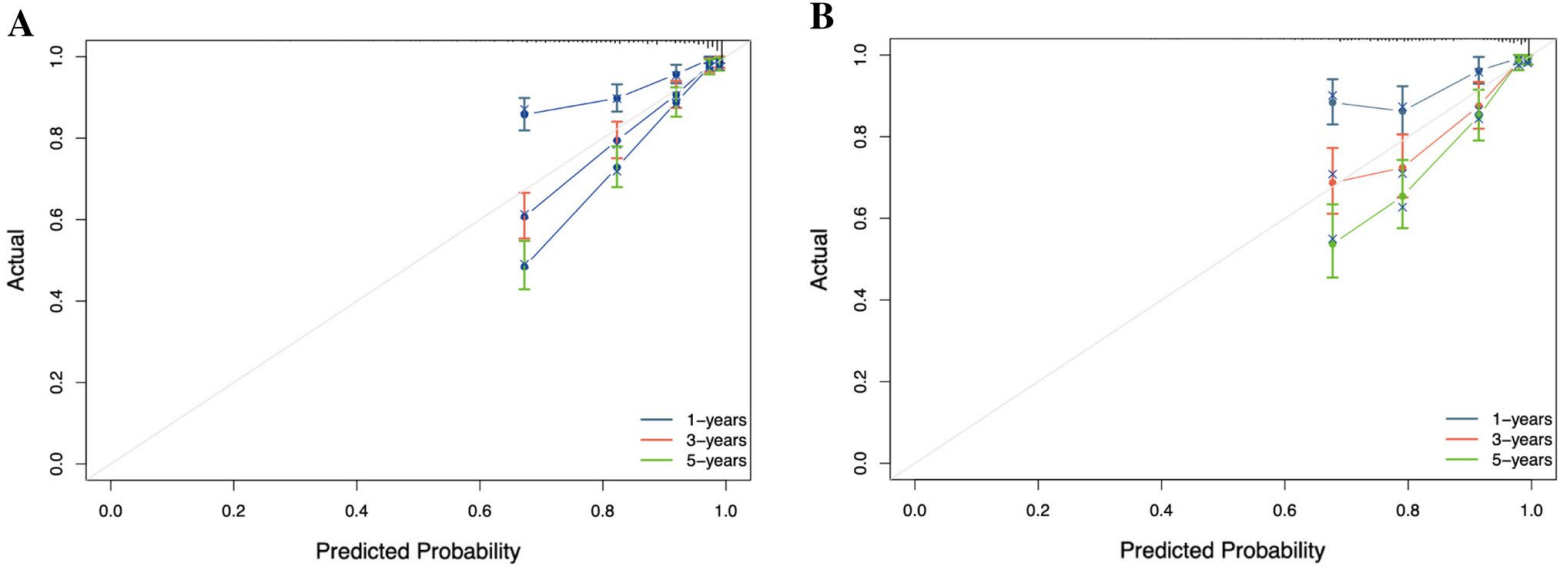
Calibration plots of the nomogram. **A** Calibration plots of 1-, 3-, and 5-year OS in the training group. **B** Calibration plots of 1-, 3-, and 5-year OS in the testing group

**Fig. 5:**
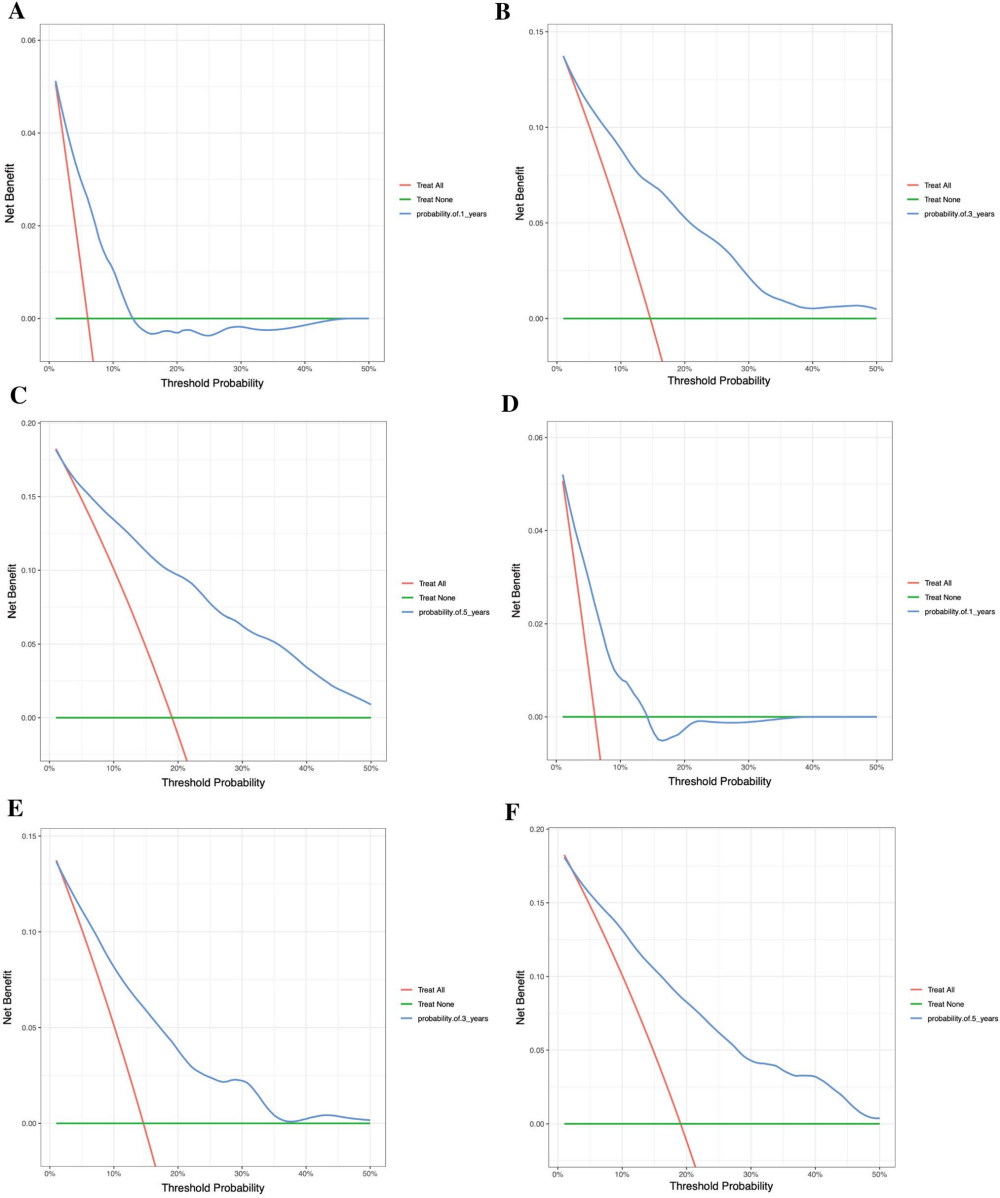
Decision curve analysis (DCA) of the nomogram. **A–C** DCA curves of 1-, 3-, and 5-year OS in the training group. **D–F** DCA curves of 1-, 3-, and 5-year OS in the testing group. The green line signifies the assumption that no patients have died, while the red line represents the supposition that all patients have died

The accuracy and applicability of the nomogram were assessed using both the training and testing groups for internal and external validation. The C-index was first used for estimation, resulting in values of 0.790 (95% CI 0.768–0.812) and 0.781 (95% CI 0.750–0.812) for the training and testing groups, respectively. In contrast, the C-index values for the conventional tumor stage were 0.718 (95% CI 0.698–0.738) and 0.724 (95% CI 0.695–0.754) in the training and testing groups, respectively. In the ROC curve analysis, the AUC values for predicting 1-, 3-, and 5-year OS were 0.764 (95% CI 0.724–0.804), 0.812 (95% CI 0.784–0.840), and 0.829 (95% CI 0.803–0.855) in the training group, and 0.730 (95% CI 0.670—0.791), 0.783 (95% CI 0.743–0.823), and 0.810 (95% CI 0.773–0.848) in the testing group, respectively (Fig. 3A, B). Comparatively, the AUC results for the conventional tumor stage were 0.718 (95% CI 0.682–0.753), 0.732 (95% CI 0.708–0.758), and 0.750 (95% CI 0.725–0.774) in the training group, and 0.713 (95% CI 0.656–0.770), 0.736 (95% CI 0.701–0.771), and 0.746 (95% CI 0.711–0.782) in the testing group for predicting 1-, 3-, and 5-year OS, respectively (Fig. 3C, D). The calibration plots also demonstrated satisfactory concordance between nomogram-predicted risk and observed risk for 1-, 3-, and 5-year OS in both the training and testing groups (Fig. 4A, B). Furthermore, the decision curves revealed that the nomogram displayed positive clinical utility in predicting OS at 1-, 3-, and 5-year intervals in both the training group (Fig. 5A–C) and the testing group (Fig. 5D–F). Overall, these results demonstrated the exceptional discriminative ability of the constructed model.

### Nomogram-Based risk stratification system

**Fig. 6:**
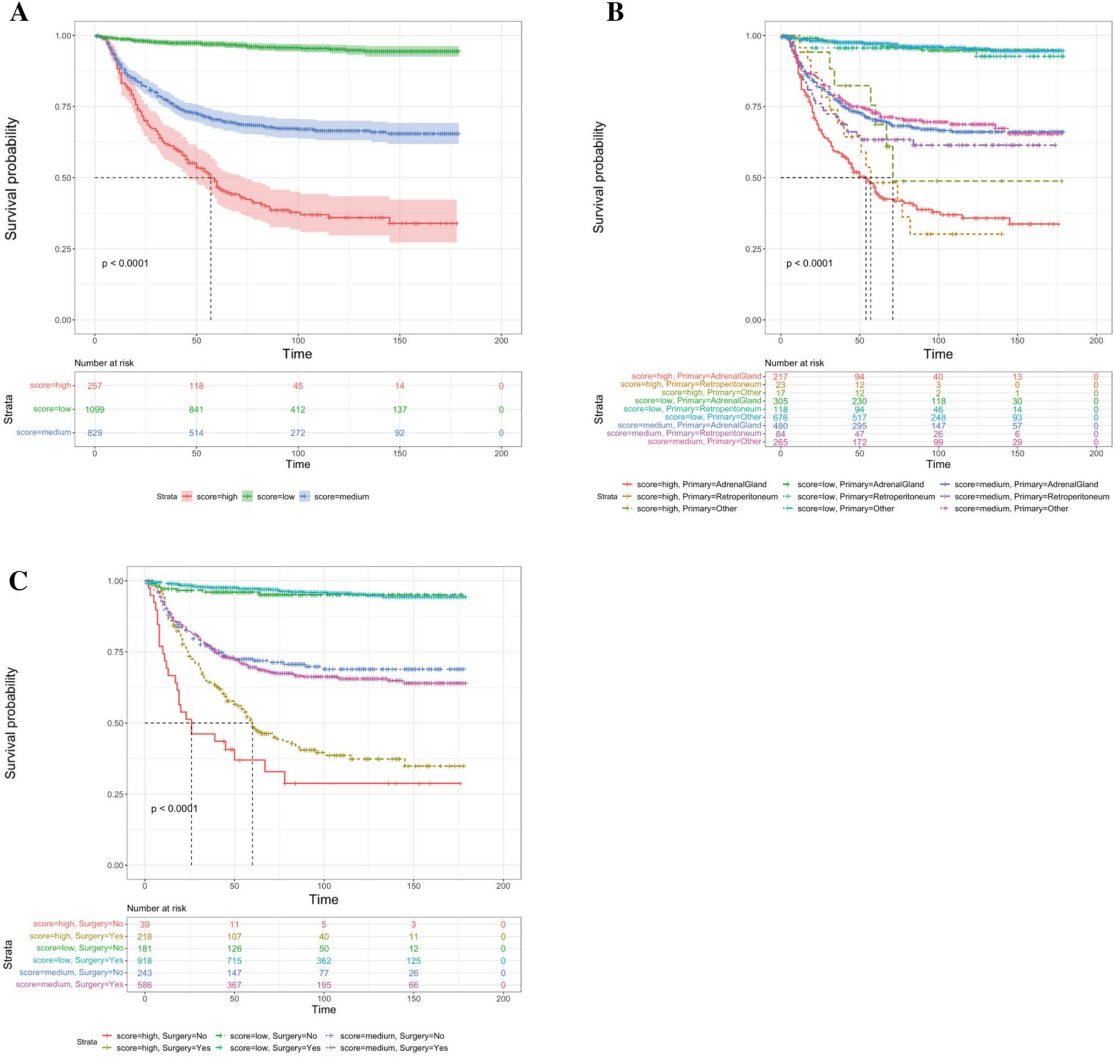
Establishment of a risk stratification system through the optimal cut-off value of the risk score. **A** Kaplan–Meier survival analysis comparing different risk groups based on nomogram scores. **B**, **C** Subgroup analysis of the primary tumor site (**B**) and surgery (**C**) according to the new risk stratification. The survival time is expressed in months

The overall prognostic score for each patient was calculated based on variables within the nomogram. Two optimal cutoff values were identified at 140 and 223 scores using X-tile software. Utilizing these thresholds, patients were stratified into low, intermediate, and high-risk groups, consisting of 1100 patients (50.3%, total score ≤ 140), 831 patients (38.0%, 140 < total score < 223), and 254 patients (11.6%, total score ≥ 223), respectively. The OS rates for the low-risk group were 98.7%, 97.4%, and 97.0% at 1, 3, and 5 years, respectively. In the intermediate-risk group, the OS rates were marginally lower at 90.0%, 77.1%, and 70.4% at 1, 3, and 5 years, respectively. However, patients in the high-risk group exhibited a substantially worse prognosis, with OS rates of merely 86.7%, 61.6%, and 46.9% at 1, 3, and 5 years, respectively (Fig. 6A).

### Subgroup analysis based on the new risk stratification

To underscore the benefits of risk stratification, we conducted an analysis of primary tumor sites and surgical outcomes across the different risk groups. Intriguingly, we observed a poorer prognosis in the retroperitoneum for the intermediate-risk group, whereas a worse prognosis was noted in the adrenal gland for the high-risk group (Fig. 6B). Moreover, we found that surgical intervention significantly enhanced the prognosis for the high-risk group (Fig. 6C).

### Web-based nomogram

We developed a web-based nomogram to predict patient outcomes (≤ 18 years) diagnosed with neuroblastoma. This accessible tool empowers physicians and patients alike to individually and visually appraise survival probability of each patient by selecting common clinical variables (https://neuroblastomanomogram.shinyapps.io/DynNomapp/).

## Discussion

The prognosis of NB is known to vary considerably based on a multitude of clinical and biological factors. However, specific biological markers may not be feasible in regions where medical resources are limited. In this study, we analyzed data from 2185 pediatric patients diagnosed with neuroblastoma between 2004 and 2015, utilizing the SEER database. We successfully identified age, chemotherapy, brain metastases, primary site, tumor stage, and tumor size as independent risk factors significantly impacting overall survival. Based on these factors, we developed a nomogram that accurately predicts 1-, 3-, and 5-year OS rates, outperforming conventional tumor staging methods in both internal and external validations. Furthermore, we established a risk classification system, derived from the nomogram model, that effectively stratifies patients into low, intermediate, and high-risk groups, facilitating early prognosis assessment. Collectively, the independent factors constituting the nomogram can be readily obtained through standard clinical practice, enhancing their broad applicability.

The prognosis of neuroblastoma patients is influenced by various key risk factors, among which the age at diagnosis plays a crucial role (Sokol et al. 2020). For stage 3 and 4 MYCN non-amplified tumors, patients under 18 months of age exhibit better event-free survival than those 18 months or older (Sokol et al. 2020). In line with this, our results also demonstrate that older patients have worse OS rates. Moreover, distant metastases is also a significant predictor of neuroblastoma patient outcomes, with a 5-year survival rate of only 19.9% for patients with brain metastases (Hu et al. 2019; Coughlan et al. 2017). Our results further corroborate that brain metastases is an independent risk factor. The primary tumor site in neuroblastoma also affects numerous aspects, including clinical and biological characteristics, event-free survival, and overall survival, with tumors in the adrenal gland associated with poorer outcomes (Vo et al. 2014). Interestingly, our findings reveal that the poorest prognosis was identified in the retroperitoneum for the intermediate-risk group, and in the adrenal gland for the high-risk group. This discrepancy may be due to differing tumor behavior and biological characteristics in these locations, although further investigation is needed to fully understand the underlying mechanisms. Furthermore, the tumor stage at diagnosis and tumor size are crucial prognostic factors for neuroblastoma patients (Brodeur and Maris 2002; Wang et al. 2022). According to our results, patients with distant tumors have a worse prognosis than those with localized and regional tumors. Additionally, larger tumors are correlated with a worse prognosis. Collectively, these independent risk factors constitute a predictive model with potential clinical utility.

The treatment strategies for neuroblastoma are multifaceted, encompassing surgery, chemotherapy, radiotherapy, retinoic acid, immunotherapy, and other supplemental treatments. Surgery plays an indispensable role in the treatment of neuroblastoma; however, it comes with its own set of challenges and potential risks, such as vascular damage or bleeding (Simon et al. 2013). Our findings demonstrate that, among high-risk patients, surgical intervention significantly improves survival outcomes. Hence, the utility of surgery may be underestimated in these high-risk patients. We recommend for the consideration of surgical intervention, wherever possible and safe, for high-risk neuroblastoma patients. In contrast to surgery, our study identified a significant association between chemotherapy and unfavorable outcomes. This could potentially be attributable to the patients receiving high-intensity chemotherapy, who were already categorized as high-risk, or it could be due to deaths related to the treatment itself.

Our study admittedly has several limitations. Firstly, the SEER database lacks some crucial prognostic variables, including MYCN amplification status, DNA ploidy, and the INSS stage. However, given that the variables included in the nomogram are readily available and easy to generalize, the nomogram predictive model based on the SEER database remains a valuable tool. Secondly, our study is retrospective, which could introduce selection bias. Therefore, further prospective clinical data are required to verify the accuracy and validity of our results.

In summary, we developed a pragmatic nomogram and risk stratification system that outperforms traditional tumor staging methods in predicting the overall survival of neuroblastoma patients. The incorporation of easily accessible clinical risk factors significantly bolsters the clinical applicability and utility of the model.

## Data Availability

The data supporting the findings of this study are sourced from the publicly available Surveillance, Epidemiology, and End Results (SEER) Program (https://seer.cancer.gov/). The authors commit to providing the raw data that supports the conclusions of this article.
